# Molecular investigation of adequate sources of mesenchymal stem cells for cell therapy of COVID‐19‐associated organ failure

**DOI:** 10.1002/sctm.20-0189

**Published:** 2020-11-25

**Authors:** Christophe Desterke, Frank Griscelli, Jusuf Imeri, Paul Marcoux, Thomas Lemonnier, Theodoros Latsis, Ali G. Turhan, Annelise Bennaceur‐Griscelli

**Affiliations:** ^1^ INSERM UMR‐S 935 and University Paris Saclay Villejuif France; ^2^ INGESTEM National IPSC Infrastructure Villejuif France; ^3^ Gustave Roussy Institute, Villejuif and Faculty of Pharmacy, Paris Descartes University Paris France; ^4^ APHP Paris Saclay Division of Hematology and University Paris Saclay Faculty of Medicine Villejuif France

**Keywords:** adult human bone marrow, embryonic stem cells, induced pluripotent stem cells, lymphocytes, mesenchymal stem cells

## Abstract

The use of mesenchymal stem cells (MSC) derived from several sources has been suggested as a major anti‐inflammation strategy during the recent outbreak of coronavirus‐19 (COVID‐19). As the virus enters the target cells through the receptor ACE2, it is important to determine if the MSC population transfused to patients could also be a target for the virus entry. We report here that ACE2 is highly expressed in adult bone marrow, adipose tissue, or umbilical cord‐derived MSC. On the other hand, placenta‐derived MSC express low levels of ACE2 but only in early passages of cultures. MSC derived from human embryonic stem cell or human induced pluripotent stem cells express also very low levels of ACE2. The transcriptome analysis of the MSCs with lowest expression of ACE2 in fetal‐like MSCs is found to be associated in particularly with an anti‐inflammatory signature. These results are of major interest for designing future clinical MSC‐based stem cell therapies for severe COVID‐19 infections.

## Graphical abstract

The authors report that mesenchymal stem cells (MSC) derived from adult sources express high levels of ACE2, which could facilitate the SARS‐Cov2 entry, compared with MSC derived from embryonic or pluripotent stem cell (iPSC)‐derived MSC. The latter population could be the most adequate sources for cell therapy of severe COVID‐19 infection, as they could be less prone to SARS‐Cov2 entry.

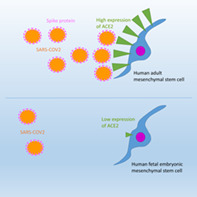

**Significance statement**

ACE2 is the receptor for COVID‐19 entry into cells. Mesenchymal stem cells (MSC) derived from several sources have been proposed as therapy for acute respiratory distress syndrome. This study focused on the expression of ACE2 in MSC and showed that the best source is MSC derived from placenta or pluripotent stem cell‐derived cells.

1

The outbreak of the new coronavirus, known as COVID‐19, has now spread from China to Europe, to the United States, and to the rest of the world, representing a major health problem for humanity.^1^ The bat SARS‐like coronavirus COVID‐19 uses the angiotensin converting enzyme receptor 2 (ACE2) to enter the cells.^2^ ACE2 is highly distributed in all adult cells, including lung, heart, kidney, liver, and endothelial cells that are then highly susceptible to infection by COVID‐19, which uses its S protein for this purpose. The high expression of ACE2 by lung alveolar cells (AT2) is one of the reasons for the major tropism of COVID‐19 for lung infection. In a subgroup of patients, COVID‐19 infection induces a strong cytokine storm and a severe disease with a macrophage activation syndrome (MAS) leading to a rapid progression to acute respiratory distress syndrome (ARDS), multiorgan failure, and death.^3^ COVID‐19‐related pneumonia is associated with lung damage and infiltration of activated pro‐inflammatory M1 macrophages associated with low IFN‐γ production by CD4 + T‐cells, as part of the virally induced immunosuppression and lymphopenia. Preliminary data suggest that the severity of COVID‐19‐associated disease may be attributable to the loss of the antiviral defense mechanism, which activates a secondary cytokine storm as a “second wave” of more tissue aggressive immunity leading to increased tissue damage. This non‐type‐1 interferon pathway implies the secretion of myeloid/macrophage derived‐cytokines, including exacerbated production of IL‐6, IL‐1β, TNF alpha, IL‐18, and GM‐CSF.

Among worldwide initiated therapeutic efforts to stop or to improve COVID‐19‐induced organ damage, the use of mesenchymal stem cells (MSC) has been proposed for their potential immune‐modulation capabilities. Regarding their anti‐inflammatory and repairing proprietary mostly by the release of secreted trophic molecules, adult MSC from bone marrow or adipose tissues have been used in the clinic to attenuate immune responses in cases of graft‐vs‐host disease (GVHD) and for several autoimmune disorders, such as severe rheumatic arhritis diseases and colitis. The beneficial effects of fetal‐like MSC, such as amniotic fluid‐derived stem cells, have been shown in experimental settings, including by our group in the cytokine storm situations induced by ischemia‐reperfusion injuries during kidney transplantation.^4^ The tolerogenic role of induced pluripotent stem cell (iPSC)‐derived MSC has also been shown in MHC‐incompatible skin transplants in mice.^5^ In the current worldwide emergency situation, several clinical trials have been authorized in China, the United States, and Israel for MSC‐based transplantation therapies of the severe ARDS syndromes caused by COVID‐19. A recent pioneering clinical study included seven patients with transfusion of umbilical cord (UC)‐derived MSC, which did not express ACE2, preventing a potential entry of the COVID‐19 in transplanted cells.^6^ The preliminary results of this trial were highly encouraging in very critically ill patients, some of whom recovered from a major COVID‐19‐induced pneumonia with disappearance of CT‐Scan lesions.^6^

Following this pioneer clinical study, several phase 1 trials have been initiated for COVID‐19 organ failure using mostly UC‐derived MSCs (https://www.who.int/ictrp/en/ and ClinicalTrials.gov). However, expression level of ACE2 by these potentially therapeutic tools can be highly detrimental if its expression is high. Currently there are no data with regard to the ACE2 receptor expression levels in MSCs of different origin used for therapy. Moreover, immune‐modulatory potency of MSCs differs depending on their organ or tissue of origin, and disease‐specific inflammatory microenvironments may influence the regulatory effect of MSCs.

We report here the results of bioinformatics and molecular analyses performed in several sources of MSCs from adult, fetal tissue, and pluripotent stem cells evaluating the expression of ACE2 and the assessment of their signaling pathways associated with their anti‐inflammatory activity. Publically available transcriptome datasets were collected from Gene Expression Omnibus (GEO) website. ACE2 expression was found to be significantly higher in MSC‐derived from adipose tissue and adult bone marrow (Figure 1A), compared with MSC‐derived from UC or placenta (*P* = .03724, Figure 1A). MSCs expressing lower levels of ACE2 were also found to be associated with lower expression of genes involved in inflammasome and immune regulation (Figure 1B,C). In contrast to adult MSCs, placental and PSC‐derived MSC with lower expression of ACE2 can potentially reduce inflammatory responses in macrophages. As can be seen in Figure 1, the molecular signature that we identified, confirms also in placenta and UC‐derived MSC, lower expression levels of indole‐amine 2,3‐dioxygenase‐1 (IDO1) which is known to induce IL‐6, IL‐1β, and TNF‐α secretion. The expression of IL‐36a, a member of the IL‐1 cytokine family contributing to the induction of pro‐inflammatory mediators (IL‐6 and chemokines) was also found to be very low. In addition, we identified a low expression of mir‐142 on placenta and umbilical cord‐derived MSC (Figure 1C). This could be of major interest for the therapeutic efficiency of these cells, as it has been reported that experimental depletion of mir‐142 in UC‐MSC induces a high expression of its target, CXCR7, which plays a critical role in the mobilization, recruitment, and function of MSCs during tissue regeneration and angiogenesis via the SDF‐1/CXCR7 axis in order to reduce hypoxia‐induced‐cell apoptosis.^7^

**FIGURE 1:**
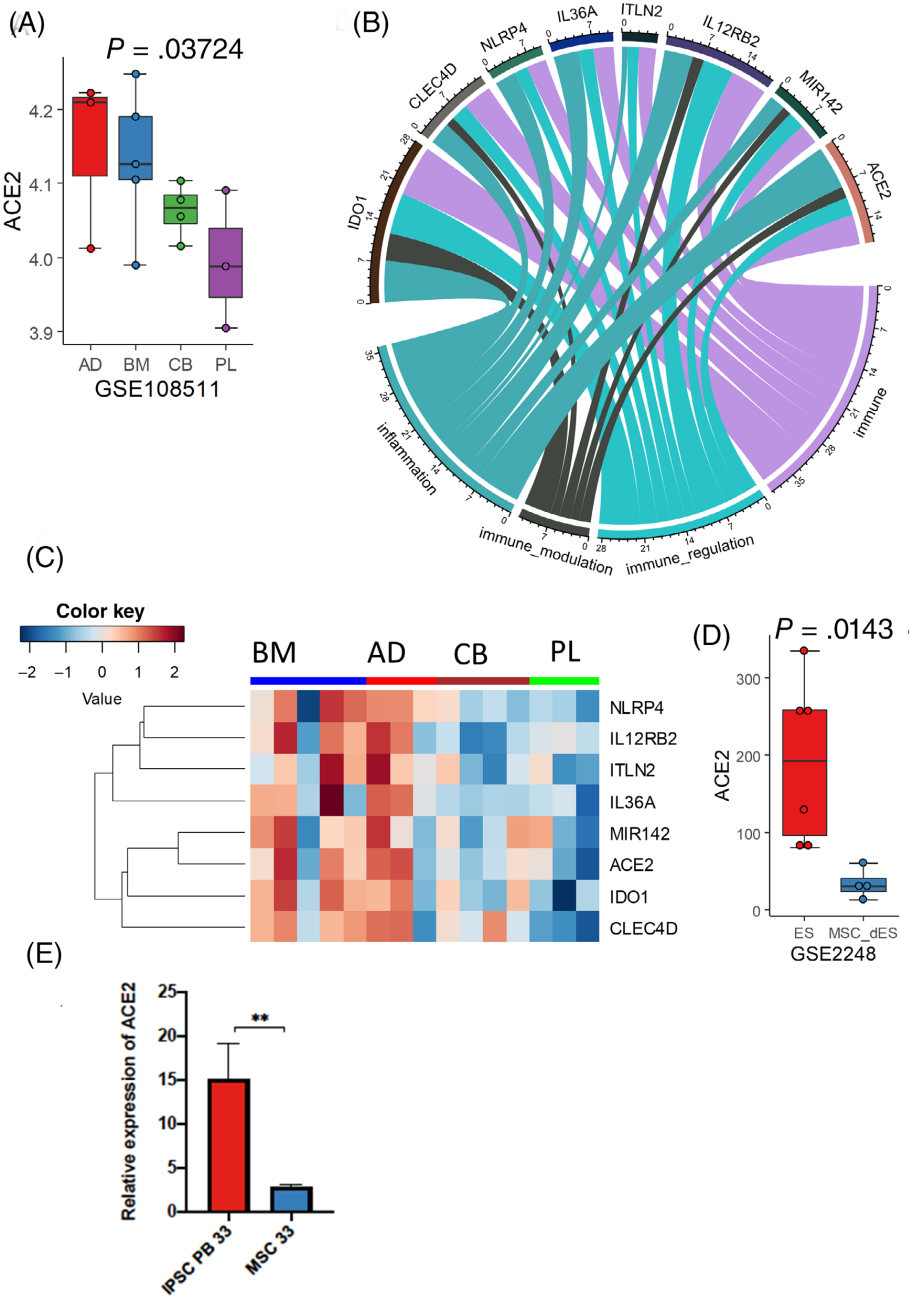
Regulation of ACE2 expression and immune signature human MSCs derived from distinct tissues. A, Expression of ACE2 in MSCs generated from distinct tissues. (p:Kruskal‐Wallis *P*‐value test); B, Circos plot of genes whose expression are correlated to ACE2 expression in human MSCs (dataset GSE108511) and identified by text mining to an immunological function (Pubmed Results). C, Heatmap expression of ACE2 signature associated with an immunity gene in human MSCs; D, Boxplot of ACE2 expression in embryonic stem cell‐derived MSCs compared with undifferentiated embryonic stem cells (GSE2248, p:Kruskal‐Wallis *P*‐value test). E, Real‐time reverse transcription‐PCR mRNA analysis of iPSCs (PB33) and MSC‐derived from the same iPSCs (passage 6). AD, adipose tissue; BM, bone marrow; CB, cord blood; PL, placenta

Interestingly, culture conditions can also impact ACE2 expression levels, as after late passages (3‐5 passages), both UC and placenta‐derived MSC were found to express higher levels of ACE2 (*P* = .006951, Supporting Information Figure S1). We then analyzed ACE2 expression levels in the human embryonic stem cell (hESC) line H1 and the MSC derived from this cell line as reported by Barberi et al.^8^ As seen in Figure 1B, although pluripotent H1 cells express very high levels of ACE2, its MSC‐derivatives express very low levels (*P* = .0143, Figure 1D). To determine if iPSCs and their MSC‐derivatives exhibit the same pattern, we used an iPSC cell line derived from bone marrow of a donor and their corresponding MSC after differentiation. As can be seen in Figure 1E, iPSC express high levels of ACE2 but the expression is reduced when these cells are induced to differentiate toward MSC. Overall, these specific characteristics suggest that fetal‐like MSCs, either derived from placenta at early passages or derived from hESC or iPSC, could be good sources of stem cell based therapy for COVID‐19‐induced organ failures. It could be also possible to select the MSC, batches used for these treatments on the basis of their ACE2 expression. Fetal‐like MSCs have already been reported to inhibit the activation of M1‐type macrophages with elicitation of M2 anti‐inflammatory polarization via TNF‐α‐mediated‐activation of cyclooxygenase‐2 (COX‐2) and TNF‐stimulated gene‐6 (TSG‐9).^9^ Therapeutic effects of MSC may thus arise from the regulation of multiple macrophage functions by targeting various cytokines simultaneously, implying that these immune‐balancing effects enable fetal‐like MSCs to be a promising therapeutic option for COVID‐19‐induced cytokine storm. However, currently there are no sufficient clinical data about the potential clinical efficacy of MSC to treat all patients with COVID‐19 disease and clinical trials using either placental or hESC/iPSC‐derived MSCs are urgently needed.

## CONFLICT OF INTEREST

The authors declared no potential conflicts of interest.

## AUTHOR CONTRIBUTIONS

A.G.T., A.B.G., F.G., C.D.: designed the research, C.D. performed bioinformaticsanalyses; T.L., J.I., T.L., P.M.: performed pluripotent stem cell cultures and PCR analyses; A.G.T., A.B.G., C.D., F.G.: wrote the paper.

## Supporting information


**Figure S1** Expression of ACE2 in materno‐fetal‐derived MSCs depending of early and late passages. Expression of ACE2 in early and late passage in human MSCs from different materno‐fetal origins: Cord, cord tissue; WJ, Wharton's jelly; CPJ, cord‐placenta junction (dataset GSE76295, p:Kruskal‐Wallis *P*‐value test)

## Data Availability

Data sharing is not applicable to this article as no new data were created or analyzed in this study.
