# Supplementary figures and images for: Molecular investigation of adequate sources of mesenchymal stem cells for cell therapy of COVID‐19‐associated organ failure
Source: Stem Cells Transl Med. 2020 Nov 25;10(4):568–71. doi: 10.1002/sctm.20-0189 (PMC7753753; doi:10.1002/sctm.20-0189)

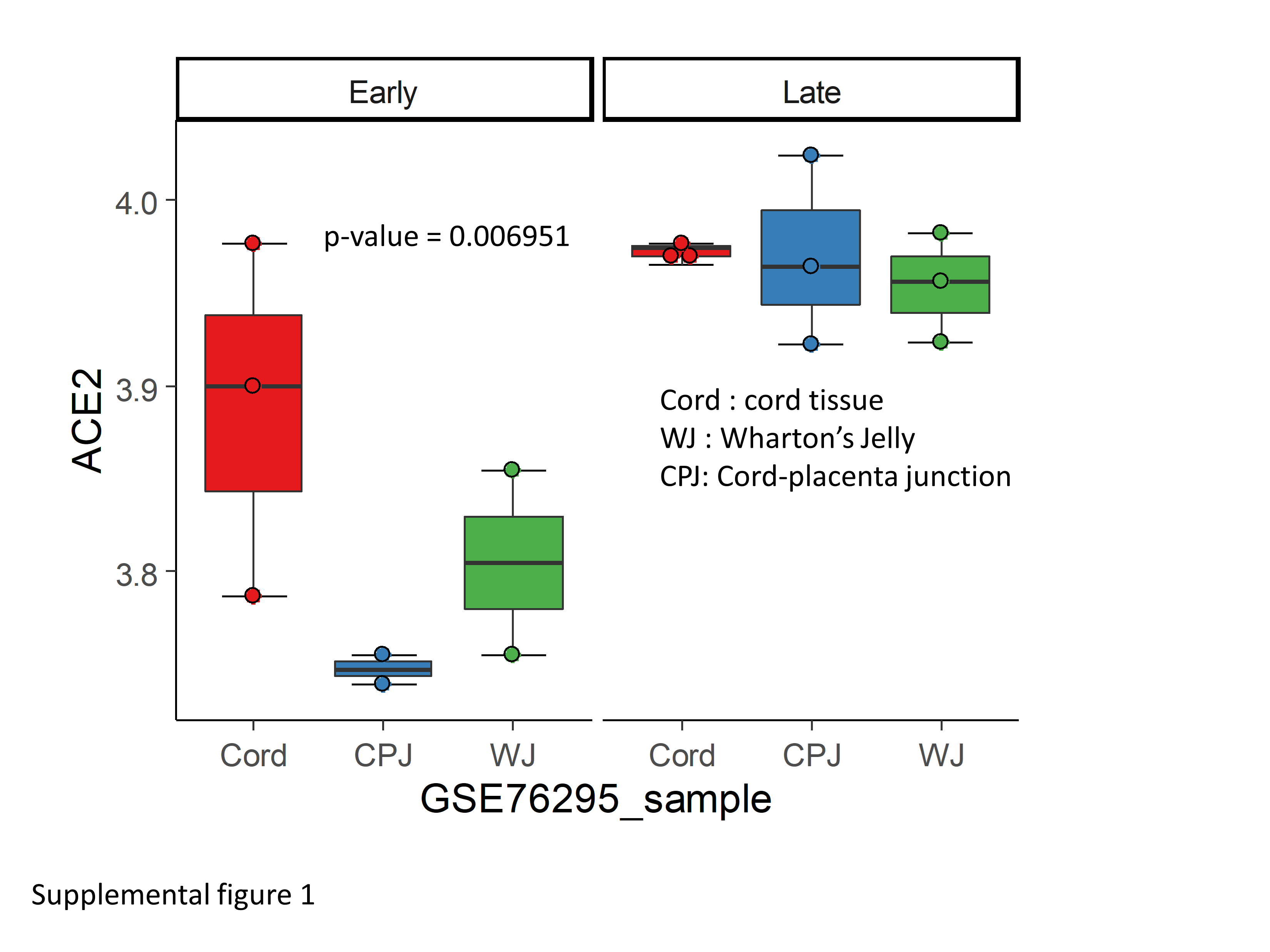

Supplement: Supplementary file 1 — Figure S1 Expression of ACE2 in materno‐fetal‐derived MSCs depending of early and late passages. Expression of ACE2 in early and late passage in human MSCs from different materno‐fetal origins: Cord, cord tissue; WJ, Wharton's jelly; CPJ, cord‐placenta junction (dataset GSE76295, p:Kruskal‐Wallis P‐value test) [file SCT3-10-568-s001.tif]
